# Association between serum neuron-specific enolase levels and short-term brain injury in pediatric febrile convulsions: A cross-sectional study

**DOI:** 10.1097/MD.0000000000046707

**Published:** 2025-12-19

**Authors:** Ling Jiang, Jing Yue, Jing Dong, Hui Zhao

**Affiliations:** a Department of Emergence, Maternal and Child Health Hospital of Hubei Province, Tongji Medical College, Huazhong University of Science and Technology, Wuhan, China.

**Keywords:** biomarker, brain injury, febrile convulsions, neuron-specific enolase

## Abstract

This study aims to investigate the association between serum neuron-specific enolase (NSE) concentrations and acute brain injury in children experiencing febrile convulsions. This cross-sectional study involved 120 children aged 6 months to 6 years with febrile convulsions from January 2021 to December 2023. Children were categorized into brain injury and non-brain injury groups based on electroencephalogram findings or neurological symptoms within 48 hours post-seizure. Serum NSE levels were measured using enzyme-linked immunosorbent assay. Logistic regression analyses were conducted to assess the association between NSE levels and brain injury, with adjustments for age, body temperature, number of convulsive episodes, and seizure duration. Subgroup analyses were performed based on convulsive episodes (<2 vs ≥2) and seizure duration (<10 minutes vs ≥10 minutes). Short-term brain injury was observed in 30% of the children. Elevated serum NSE levels were significantly associated with brain injury (odds ratio [OR]: 4.2, 95% confidence interval [CI]: 2.1–8.4, *P* < .001 in univariate analysis; OR: 3.8, 95% CI: 1.9–7.6, *P* < .001 in multivariate analysis). The association was particularly strong in children with ≥2 convulsive episodes (OR: 4.5, 95% CI: 1.9–10.7, *P* = .001) and prolonged seizures (≥10 minutes, OR: 6.0, 95% CI: 2.3–15.8, *P* < .001). Elevated serum NSE levels were associated with acute electroencephalogram or imaging indicators of brain injury in children with febrile convulsions, especially in those with repeated or prolonged seizures.

## 1. Introduction

Febrile convulsions (FC) are among the most frequent neurological emergencies in pediatric practice, affecting around 2% to 5% of children between the ages of 6 months and 5 years.^[1]^ These seizures typically occur during a febrile illness and are characterized by generalized convulsions, often accompanied by a rapid increase in body temperature.^[2]^ Although most febrile convulsions are benign and self-limiting, a subset of children may experience short-term brain injury, which may result in neurological complications, including cognitive impairment, behavioral changes, and increased risk of developing epilepsy.^[3]^

Short-term brain injury in the context of febrile convulsions is often assessed through clinical symptoms, electroencephalogram (EEG) abnormalities, and neuroimaging studies such as cranial magnetic resonance imaging (MRI).^[4]^ Previous studies have shown that prolonged seizure duration and multiple convulsive episodes are significant risk factors for brain injury.^[5]^ However, identifying children at higher risk of brain injury remains challenging, necessitating the exploration of reliable biomarkers.

Neuron-specific enolase (NSE) serves as a glycolytic enzyme that is specifically found in neurons and certain neuroendocrine cells.^[6]^ Increased concentrations of NSE in the serum have been observed in a range of neurological disorders, such as brain injuries caused by trauma, stroke, and diseases associated with neurodegeneration, where it serves as a marker of neuronal damage.^[7]^ Whether it similarly reflects acute injury in children with febrile convulsions remains unclear—although some studies have suggested a possible association.^[8]^

The primary objective of this study was to investigate the association between serum NSE levels and short-term brain injury in children with febrile convulsions. We hypothesized that elevated serum NSE levels would be significantly associated with an increased risk of short-term brain injury, particularly in children with more severe seizure characteristics such as multiple convulsive episodes and prolonged seizure duration. Additionally, we aimed to explore whether this association varied across different clinical subgroups.

## 2. Methods

### 2.1. Study design and population

This cross-sectional study took place in the Department of Pediatrics at our hospital from January 2021 to December 2023. During this period, all children aged 6 months to 6 years who presented with fever and acute seizures were consecutively screened for eligibility. The research protocol was examined and authorized by the Ethics Committee of our hospital. Written informed consent was acquired from the parents or legal guardians of all children involved in the study.

### 2.2. Sample size calculation

Prior to patient recruitment, we performed a sample size estimation based on the primary objective: to detect an association between serum NSE (continuous predictor) and short-term brain injury (binary outcome). Assuming a 30% prevalence of brain injury, an odds ratio of 3.0 per 10 ng/mL increase in NSE, and a standard deviation of 7.0 ng/mL, we estimated that a minimum of 105 participants would be required to achieve 80% power at α = 0.05 (2-sided) using logistic regression. To allow for a predicted 10 % exclusion or missing-data rate, we planned to screen approximately 135 children. Ultimately, 135 patients were screened and 120 fulfilled all inclusion/exclusion criteria, consistent with our planning and sufficient for the primary analysis.

### 2.3. Participants and eligibility criteria

Children were included if they fulfilled the diagnostic standards for febrile seizures according to the Specialist Agreement on the Diagnosis, Therapy, and Handling of Febrile Seizures (2016),^[9]^ which includes a clear history of fever (defined as a body temperature ≥38.4°C (101.0°F), measured rectally or via axillary thermometry), clinical manifestations of convulsions, and exclusion of other causes of seizures. Both first-time and recurrent cases were included. Participants were excluded if they had preexisting focal neurological deficits or neurodevelopmental disorders neonatal febrile convulsions, congenital heart disease, genetic disorders, or other congenital abnormalities. Children with meningitis, encephalitis, epilepsy, phenylketonuria, or other neurological diseases were also excluded. Additionally, children with comorbidities involving heart, liver, kidney, or other organ dysfunction, or those using medications that could affect seizure occurrence or brain injury assessment were excluded from the study.

### 2.4. Data collection

Clinical information was gathered for all participants, including their age and gender, body temperature at presentation, number of convulsive episodes, duration of seizures, and type of seizures (simple vs complex). For each child, a detailed patient background, clinical assessment observations, and diagnostic evaluation outcomes were recorded. For EEG, short-term brain injury was identified by the presence of focal or diffuse slowing, or focal interictal spikes or sharp waves within 48 hours after seizure onset. Generalized spike-and-wave patterns were explicitly excluded from our definition to maintain specificity.

We used Glasgow Coma Scale (GCS) to objectively quantify levels of lethargy and altered mental status. A GCS score below a predetermined threshold was considered as evidence of impaired consciousness and potential brain injury. This threshold was determined based on established clinical guidelines and our study’s specific criteria to ensure it accurately reflected significant alterations in mental status.

When clinically indicated, cranial MRI was performed to further assess for structural brain injury. The presence of specific MRI findings, such as edema, hemorrhage, or other structural abnormalities consistent with acute neuronal injury, was used to confirm the presence of short-term brain injury.

Short-term brain injury in children with febrile convulsions was defined based on a combination of clinical and neurophysiological assessments. The diagnostic approach involved 2 stages: children were initially screened for potential brain injury if they presented with EEG abnormalities, or lethargy, or altered mental status. For children who screened positive, further assessments including GCS evaluation and cranial MRI were conducted. Based on the existence or nonexistence of short-term brain injury, children with febrile convulsions were categorized into 2 groups: the brain injury group and the non-brain injury group. All EEG and MRI recordings were interpreted by 2 board-certified pediatric neurologists and 1 radiologist who were blinded to serum NSE concentrations and final group assignment; any disagreements were resolved by an additional independent pediatric electroencephalographer or neuroradiologist (as appropriate) who was equally unaware of NSE results and grouping. EEG definitions: Diffuse slowing: background activity showing ≥50% theta (4–8 Hz) or delta (1.5–4 Hz) waves with amplitude ≥20 µV, loss of the posterior-dominant alpha rhythm, and duration ≥1 minute. Focal slowing: localized, continuous or recurrent delta (1.5–4 Hz) activity ≥20 µV lasting ≥10 seconds and occupying at least 1 electrode derivation, with ≥30% power increase compared with the homologous contralateral region. GCS threshold: Altered consciousness was defined as a GCS score ≤14 measured within the first 30 minutes after seizure cessation.

### 2.5. Measurement of serum NSE

Venous blood samples (5 mL) were obtained from each participant within the first 24 hours after admission. Blood was collected in vacutainers, allowed to clot for 30 minutes. The sample was then centrifuged at 3000 revolutions per minute for a duration of 10 minutes. Following this, the serum was isolated and maintained at −80°C until further analysis. Serum NSE levels were determined by the enzyme-linked immunosorbent assay (Wuhan Fine Biotech Co., Ltd.). The enzyme-linked immunosorbent assay method primarily detects the γγ isoenzyme of NSE. The kits were used strictly according to the manufacturer’s protocol. All samples were analyzed in duplicate, and the interassay and intraassay coefficients of variation were maintained below 10%, ensuring the precision of our results.

### 2.6. Statistical analysis

The data analysis was conducted utilizing SPSS version 26.0. Continuous variables are expressed as mean ± standard deviation, while categorical variables are described using frequencies or percentages. Comparisons of continuous variables were made using independent samples *t* tests or Mann–Whitney *U* tests (for non-normally distributed data). Categorical variables were analyzed using chi-square tests or Fisher exact tests as appropriate. Additionally, univariate analysis was carried out to compare clinical characteristics and serum NSE levels between the brain injury group and the non-brain injury group. A multivariate logistic regression analysis was performed to evaluate the independent relationship between serum NSE levels and short-term brain injury. Taking into account possible confounding factors like age, body temperature, number of convulsive episodes, and duration of seizures. Multivariate model: binary logistic regression with forced entry of the following a priori defined covariates: serum NSE (continuous, per 10 ng/mL increase); number of convulsive episodes (continuous, count); seizure duration (binary, ≥10 minutes vs <10 minutes); age (continuous, per 1-year increase); body temperature at presentation (continuous, per 1°C increase). Odds ratios and 95% confidence intervals (CI) are reported; all 5 covariates were retained regardless of statistical significance. The multivariate logistic regression model included all 120 participants with complete data for all covariates.

Subgroup analyses were performed based on the number of convulsive episodes (<2 vs ≥2) and duration of seizures (<10 minutes vs ≥10 minutes) to further investigate the connection between serum NSE concentrations and short-term brain injury in different clinical scenarios. A 2-sided *P*-value <.05 was regarded as indicating statistical significance.

## 3. Results

### 3.1. Study population’s baseline features

Of the 135 children initially screened with febrile convulsions, 15 were excluded: 7 for preexisting neurodevelopmental disorders, 5 for incomplete blood sampling, and 3 for refusal of EEG. Consequently, 120 children (aged 6 months–6 years) with complete data were enrolled in the final analysis. Their mean age was 3.1 ± 1.2 years and 65 % were boys. The mean body temperature at presentation was 39.3 ± 0.6°C. Among the patients, convulsions were classified as generalized in 76 cases and focal in 44 cases. Of these, 28 patients with focal seizures were in the brain injury group, while 16 were in the non-brain injury group. Overall, 40 patients experienced complex seizures. Within this group, 26 patients belonged to the brain injury group and 14 to the non-brain injury group. The number of convulsive episodes varied between 1 and 4, having an average of 1.7 ± 0.9 episodes per child. The mean duration of seizures was 7.6 ± 4.2 minutes. Short-term brain injury was identified in 36 out of 120 children with febrile convulsions, representing a frequency of 30%. As summarized in Table 1, children with brain injury had more convulsive episodes (mean 2.1 vs 1.5), longer seizures (10.4 vs 6.2 minutes), higher rates of complex (72% vs 17%) and focal (78% vs 19%) seizures, and markedly elevated serum NSE levels (34.2 vs 20.5 ng/mL) compared with those without injury, whereas age, sex and initial temperature were similar between groups. Children in the brain injury group had significantly higher serum NSE levels in contrast to those in the non-brain injury group (*P* < .001). Among the 36 children in the brain injury group, 12 (33.3%) underwent cranial MRI based on clinical indications (e.g., prolonged seizures or focal neurological deficits). Of these, 8 (66.7%) showed acute structural abnormalities, including hippocampal edema (n = 6) and cortical hyperintensity (n = 2). Additionally, 16 patients had EEG abnormalities, and 11 patients exhibited a GCS score below the threshold. The brain injury group exhibited a higher number of convulsive episodes and longer seizure durations (Table 1).

**Table 1:** Comparison of clinical characteristics and serum NSE levels between brain injury and non-brain injury groups.

Characteristic	Brain injury group (n = 36)	Non-brain injury group (n = 84)	*P*-value
Age (yr)	3.2 ± 1.1	3.0 ± 1.3	.34
Boy (n, %)	25 (69%)	53 (63%)	.48
Body temperature (°C)	39.5 ± 0.5	39.2 ± 0.6	.08
Number of convulsive episodes	2.1 ± 1.0	1.5 ± 0.8	.002
Duration of seizures (min)	10.4 ± 5.1	6.2 ± 3.4	<.001
First-time febrile seizure	20 (56%)	49 (58%)	.27
Serum NSE (ng/mL) after admission	34.2 ± 7.6	20.5 ± 5.8	<.001
Complex febrile seizures	26 (72%)	14 (17%)	<.001
Seizures with focal features	28 (78%)	16 (19%)	<.001
Convalescent serum NSE (ng/mL)	18.1 ± 3.2	–	–

NSE = neuron-specific enolase.

### 3.2. Association between NSE and brain injury in FC patients

In univariate analysis, each 10 ng/mL increase in serum NSE was linked to a 4.2 times higher risk of brain injury (95% CI: 2.1–8.4, *P* < .001). This relationship continued to be significant even after adjustment for number of convulsive episodes and duration of seizures, with a likelihood ratio of 3.8 (95% CI: 1.9–7.6, *P* < .001, Table 2).

**Table 2:** Association between NSE and short-term brain injury in FC patients.

Variable	Univariate analysis		Multivariate analysis	
	OR (95% CI)	*P*-value	OR (95% CI)	*P*-value
Serum NSE (per 10 ng/mL)	4.2 (2.1–8.4)	<.001	3.8 (1.9–7.6)	<.001
Number of convulsive episodes	3.0 (1.5–5.9)	.002	2.7 (1.3–5.6)	.007
Duration of seizures (≥10 min)	3.5 (1.6–7.6)	.003	3.2 (1.4–7.3)	.006
Age (per yr)	0.9 (0.7–1.2)	.45		
Body temperature (per °C)	1.2 (0.8–1.7)	.48		

Multivariate model adjusted for the 5 covariates listed in 2.6: serum NSE (per 10 ng/mL), number of convulsive episodes, seizure duration (≥10 min), age, and body temperature. All 120 participants were included in the multivariate model; no missing data were observed.

CI = confidence interval, FC = febrile convulsions, NSE = neuron-specific enolase, OR = odds ratio.

### 3.3. Subgroup analysis based on seizure characteristics

The results indicate that the association between elevated serum NSE levels and short-term brain injury is particularly strong in children with multiple convulsive episodes (≥2) and prolonged seizure durations (≥10 minutes). In these subgroups, elevated NSE levels are strongly linked to a higher likelihood of brain injury. In contrast, the association is weaker or not significant in children with fewer convulsive episodes (*P* = .65), shorter seizure durations (*P* = .84). The subgroup analysis of body temperature and age did not reveal a differential association. This suggests that NSE levels may be a more reliable biomarker for brain injury in children with more severe seizure characteristics (Table 3). The forest plot for subgroup analysis of the association between NSE and short-term brain injury was presented in Figure S1, Supplemental Digital Content, https://links.lww.com/MD/Q942.

**Table 3:** Subgroup analysis of the association between NSE and short-term brain injury.

Subgroup characteristic	Subgroup category	n (%)	Brain injury cases (%)	Mean serum NSE (ng/mL)	OR, 95% CI	*P*-value
Number of convulsive episodes	<2 episodes	70 (58.3)	15 (21.4)	22.3 ± 6.1	1.2 (0.5–2.8)	.65
	≥2 episodes	50 (41.7)	21 (42.0)	35.4 ± 8.2	4.5 (1.9–10.7)	.001
Duration of seizures	<10 min	80 (66.7)	16 (20.0)	21.5 ± 5.9	1.1 (0.4–2.9)	.84
	≥10 min	40 (33.3)	20 (50.0)	38.2 ± 9.1	6.0 (2.3–15.8)	<.001
Age	<3 yr	60 (50.0)	18 (30.0)	23.1 ± 6.5	2.1 (0.9–4.8)	.08
	≥3 yr	60 (50.0)	18 (30.0)	36.8 ± 7.8	3.0 (1.3–6.9)	.22
Body temperature	<39.5°C	65 (54.2)	17 (26.2)	22.8 ± 6.3	1.5 (0.6–3.5)	.38
	≥39.5°C	55 (45.8)	19 (34.5)	37.5 ± 8.5	2.8 (1.2–6.7)	.27

CI = confidence interval, NSE = neuron-specific enolase, OR = odds ratio.

## 4. Discussion

The objective of the study was to explore the association between serum NSE levels and short-term brain injury in children with FC. The findings revealed that elevated serum NSE Levels showed a significant association with a heightened risk of short-term brain injury, particularly in children with multiple convulsive episodes (≥2) (*P* = .001) and prolonged seizure durations (≥10 minutes) (*P* < .001). This association was less evident in children with fewer convulsive episodes (*P* = .65) or shorter seizure durations (*P* = .84).

Elevated NSE reflects neuronal membrane disruption; its release into the bloodstream within hours after a seizure makes it a plausible proxy for acute insult.^[10,11]^ In the context of febrile convulsions, NSE levels may reflect neuronal damage caused by the seizures.^[12]^ Wang et al and Pratamastuti et al have already reported a positive cross-sectional association between NSE and brain injury in children with febrile convulsions^[13]^; our prospective design now quantifies this relationship as a dose–response (per 10 ng/mL) and restricts the outcome to acute EEG/MRI changes, supporting NSE as a continuous rather than dichotomous indicator of early neuronal stress. These findings collectively suggest that NSE may be a reliable marker for neuronal damage in this population.

Negative studies obtained blood ≥24 hours after seizure onset, a time-window in which NSE has already returned to baseline in adult TBI cohorts^[14,15]^; furthermore, they grouped simple and complex febrile seizures together, diluting any true biological gradient.^[16]^ By sampling within 6 hours of seizure cessation and by defining acute injury with standardized EEG/MRI criteria, we captured the early release phase of NSE and reduced phenotypic heterogeneity—thereby maximizing both biological signal and the observable difference between severity groups. Our brain injury case group included a relatively higher proportion of patients with focal seizures and complex seizures.^[17]^ Patients with focal or complex seizures often exhibit more pronounced brain injuries compared to those with generalized or simple seizures. As a result, a stronger association was observed in our study.

However, our study extends the existing literature by providing more detailed subgroup analyses. We found that the association between elevated NSE levels and brain injury was particularly strong in children with multiple convulsive episodes or prolonged seizures. This is an important addition to the current understanding, as previous studies did not specifically explore the relationship in these subgroups.

Repeated or prolonged seizures aggregate excitotoxic, hypoxic and metabolic stress that amplify neuronal injury,^[18–20]^ explaining the stepwise NSE increase we observed; consistent with this mechanism, the FEBSTAT cohort reported hippocampal sclerosis and frequent focal EEG slowing in children with febrile status epilepticus.^[21,22]^ This is supported by studies showing that the duration and frequency of seizures are significant predictors of brain injury.^[23]^ The pathophysiological mechanisms underlying brain injury in these subgroups may involve increased excitotoxicity, oxidative stress, and inflammatory responses, which are known to contribute to neuronal damage.^[24,25]^ Excitotoxicity occurs when excessive glutamate release results in excessive activation of NMDA receptors, causing a surge of calcium ions and ensuing neuronal damage.^[26]^ These mechanisms may explain why children with more severe seizure characteristics have higher NSE levels and a greater risk of short-term brain injury.^[27]^ Briefly, seizure-driven neuronal membrane disruption allows rapid, passive efflux of the cytosolic protein NSE into blood, offering a biologically plausible marker of acute neuronal injury. The strong association between elevated NSE levels and brain injury in these subgroups suggests that NSE could serve as a valuable clinical marker for identifying high-risk patients.^[28]^ Early identification of such patients may facilitate timely interventions to reduce the likelihood of long-term neurological complications.

Although GFAP and S-100B show superior sensitivity to NSE in mild traumatic brain injury, their release kinetics and diagnostic thresholds in febrile convulsion-related cytotoxic injury remain uncharted.^[29,30]^ This may be because more work is needed to confirm the specificity and sensitivity of NSE as a biomarker for brain injury. In addition, the complexity of NSE level changes in different clinical settings also contributes to this situation. Future studies should compare NSE with other emerging biomarkers. This will help us better understand the advantages and limitations of these biomarkers in pediatric populations.

The strong association between elevated NSE levels and brain injury in children with multiple convulsive episodes or prolonged seizures suggests that NSE could serve as a valuable clinical marker for identifying high-risk patients. Early identification of such patients may facilitate timely interventions aimed at reducing the long-term risk neurological sequelae. Furthermore, our subgroup analysis indicates that the relationship between NSE levels and brain injury is more pronounced in children with more severe seizure characteristics, highlighting the need for close monitoring and potential therapeutic interventions in this subgroup.

Our research is subject to several limitations that should be acknowledged. First, the cross-sectional nature of the study restricts our capacity to determine a causal link between increased NSE levels and brain injury. Longitudinal studies will be essential in the future to clarify the chronological relationship between these factors. Second, although the overall sample size was sufficient for the primary analysis, the exploratory subgroup comparisons were under-powered, so null findings in such small subgroups may reflect type II error. Confirmation in larger, multicentre cohorts is therefore needed. Third, NSE was assayed only once, precluding assessment of its temporal profile or peak concentration; repeated sampling would provide stronger evidence on the duration and magnitude of neuronal injury. Fourth, because cranial MRI was performed only in 12 of 36 children with clinically suspected injury, this selective imaging strategy may introduce selection bias and could either over- or underestimate the true prevalence of structural brain damage. Lastly, we did not routinely measure serum NSE levels in febrile patients without seizures. To overcome the inherent constraints of our cross-sectional analysis, future studies should adopt a prospective, multicentre design that enrolls children at the time of their first febrile seizure and follows them sequentially. Serial sampling of serum NSE within the first 6 to 24 hours, together with repeated EEG and standardized neuroimaging at predefined intervals, would clarify the temporal profile and peak concentration of NSE release while minimizing detection bias. Importantly, the inclusion of “fever-only” controls—children with comparable fever but no seizure—will distinguish seizure-specific neuronal injury from the systemic effects of high temperature alone. Finally, long-term neurodevelopmental follow-up (≥2 years) is needed to determine whether early NSE elevations predict later cognitive, behavioral, or epileptic sequelae, thereby establishing the true prognostic value of this biomarker.

## 5. Conclusion

In this cross-sectional study, elevated serum NSE levels were associated with short-term electroencephalographic or imaging indicators of brain injury in children with febrile convulsions, particularly among those with multiple or prolonged seizures.

## Author contributions

**Conceptualization:** Ling Jiang, Jing Yue, Jing Dong, Hui Zhao.

**Data curation:** Ling Jiang, Jing Yue, Jing Dong, Hui Zhao.

**Investigation:** Ling Jiang, Jing Yue, Jing Dong.

**Methodology:** Jing Yue, Jing Dong.

**Software:** Jing Dong.

**Supervision:** Hui Zhao.

**Validation:** Ling Jiang, Hui Zhao.

**Visualization:** Jing Yue.

**Writing – original draft:** Ling Jiang, Jing Yue, Jing Dong, Hui Zhao.

**Writing – review & editing:** Ling Jiang, Hui Zhao.

## Supplementary Material


