# Supplementary material for: Association between serum neuron-specific enolase levels and short-term brain injury in pediatric febrile convulsions: A cross-sectional study
Source: Medicine (Baltimore). 2025 Dec 19;104(51):e46707. doi: 10.1097/MD.0000000000046707 (PMC12727293; doi:10.1097/MD.0000000000046707)
Supplement: Supplementary file 1 [file medi-104-e46707-s001.docx]

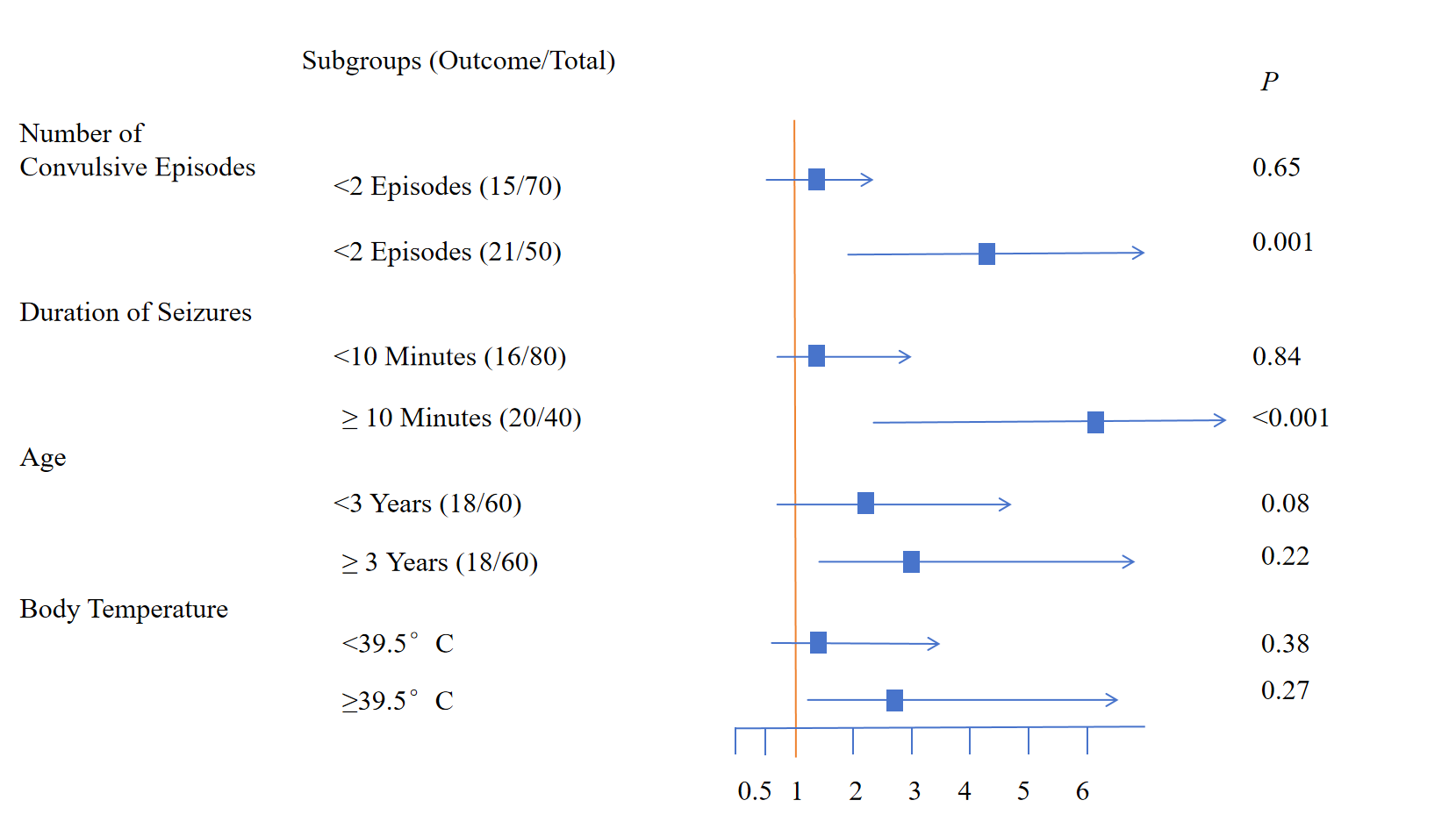


Supplementary Figure.1: Forest plot for s**ubgroup analysis of the association between NSE and short-term brain injury**
